# A scientist’s take on scientific evidence in the courtroom

**DOI:** 10.1073/pnas.2301839120

**Published:** 2023-10-02

**Authors:** Thomas D. Albright

**Affiliations:** ^a^The Salk Institute for Biological Studies, La Jolla, CA 92037

## Abstract

Scientific evidence is frequently offered to answer questions of fact in a court of law. DNA genotyping may link a suspect to a homicide. Receptor binding assays and behavioral toxicology may testify to the teratogenic effects of bug repellant. As for any use of science to inform fateful decisions, the immediate question raised is one of credibility: Is the evidence a product of valid methods? Are results accurate and reproducible? While the rigorous criteria of modern science seem a natural model for this evaluation, there are features unique to the courtroom that make the decision process scarcely recognizable by normal standards of scientific investigation. First, much science lies beyond the ken of those who must decide; outside “experts” must be called upon to advise. Second, questions of fact demand immediate resolution; decisions must be based on the science of the day. Third, in contrast to the generative adversarial process of scientific investigation, which yields successive approximations to the truth, the truth-seeking strategy of American courts is terminally adversarial, which risks fracturing knowledge along lines of discord. Wary of threats to credibility, courts have adopted formal rules for determining whether scientific testimony is trustworthy. Here, I consider the effectiveness of these rules and explore tension between the scientists’ ideal that momentous decisions should be based upon the highest standards of evidence and the practical reality that those standards are difficult to meet. Justice lies in carefully crafted compromise that benefits from robust bonds between science and law.

                        *‘Bridgeport?’ said I. ‘Camelot,’ said he.*

                                 Mark Twain (1).

Science and law have long intertwined roots as disciplines commonly devoted to rational choice. Legislative and executive branches of our government routinely turn to scientific discoveries to guide the development of laws, policies, and regulations to serve and protect members of society. Similarly, our courts rely heavily upon scientific knowledge to make informed decisions about disputes over matters such as ownership, causality, and responsibility. In this essay, I focus on the role of science in courtroom litigation, as this is the area of law that places the greatest demands on scientific evidence.

I acknowledge at the outset that this is not new ground, but it is ground infrequently traveled by laboratory scientists. The complex and sometimes contentious relationship between science and law is the subject of countless law review articles and amicus briefs, not to mention a comprehensive five-volume treatise by law professors (2). Prominent institutions of science long ago trumpeted the standards of science as a natural model for evaluation of scientific evidence by the courts:

“The scientific community’s well-established criteria and institutional mechanisms for evaluating the validity of scientific assertions provide courts with clear and understandable guidance on how they can rationally and consistently evaluate scientific evidence. Courts should admit scientific evidence only if it conforms to scientific standards and is derived from methods that are generally accepted by the scientific community as valid and reliable (3).”

Though this counsel serves as an ideal, much of the landscape of the scientific enterprise familiar to most practitioners—a “generative adversarial” process that yields ever-growing knowledge and certainty—is distorted or compromised by the “terminal adversarial” approach to truth employed by our courts. In the following discussions, I introduce a number of issues that arise from this particular use of science. Some of these are shared generally—or at least superficially—with the practice of scientific research, including assessment of the trustworthiness of evidence, the degree to which it reflects the consensus of the day, and the probability that it provides a correct solution to a problem in the real world. Other issues are unique to the legal context, including the procedure by which evidence is communicated to the court, and the exigence and resoluteness of decisions. My goal is to highlight areas at the intersection of science and courtroom law that raise interesting and sometimes unresolved questions—particularly when seen from the perspective of the scientific community—and would benefit from greater interdisciplinary collaboration.

## Specialized Knowledge in the Courtroom

Scientific investigation and courtroom litigation have many notable differences in concept, history, and procedure (4, 5), but they both operate through a process of “drawing inferences from evidence to test hypotheses and justify conclusions” (6). Because evidence is the crux of rational decisions, both disciplines have adopted standards to establish its trustworthiness. In the sciences, those standards are defined by the scientific method and policed by peer review, which ensures that evidence is based on valid methods and is carefully interpreted to develop and test hypotheses, advance theoretical perspectives, and inspire sound inventions.

Analogously, our justice system is a recipient of evidence from many sources intended to support one or another hypothesis before the court. The diversity of evidence received means that it may, or may not, fall within the common experience of the trier of fact. In some cases, the interpretation of evidence is patently obvious (the weapon was in the suspect’s bag), but in other cases (the shampoo contains a carcinogen), additional technical information is needed to guide the trier of fact (7). That guidance is generally provided by an expert witness whose “scientific, technical, or other specialized knowledge” will help the judge or jury understand the evidence. The court’s use of this expert approach is defined by a series of rulings and legislative actions that impose quality standards for admission of evidence, the primary goal of which is to ensure that the information is trustworthy. The trial judge is the designated “gatekeeper,” who oversees the evaluation of expert testimony in the context of existing rules (8), ultimately determining whether it can be used as a sound basis for probabilistic inference.

In many instances, the expert is someone who has gained subject-matter expertise through unique first-hand experiences in a particular knowledge domain. Suppose, for example, that the court wishes to determine who is at fault for a ship collision in the harbor. The testimony of a skilled tugboat captain might serve as evidence for this determination, as she is likely to possess accurate information about the probabilities of relevant events. This expert testimony may come in the form of a diagnosis (the larger ship’s pilot failed to see the smaller vessel), or it may come as a thesis on navigational principles [“framework testimony” (9, 10)], which the jury can apply to facts of the case. Either way, the question of admissibility is straightforward because the subject-matter expert is an embodiment of the evidence acquired through her own personal experience guiding massive watercraft through tight spaces. The same logic holds for dry cleaners, foresters, and taxi drivers, or any other individual who possesses a desired type of specialized knowledge acquired through personal experience.

## Uses of Scientific Knowledge in Courtroom Litigation

Fueled by the expansive growth of science over the past century, scientific investigation and courtroom litigation now commonly converge: Scientific evidence is brought before the court to help resolve questions of fact. Broadly considered, those questions are of four types, which science is generally well equipped to address:

*1) Who broke the law?*

This is the forensic question, in which a science-based reconstruction of events is used to determine human responsibility for a criminal act. A valid demonstration that DNA found at a crime scene has a high probability of association with a suspect is a common form of scientific evidence introduced in litigation.

*2) Who is liable for loss or harm?*

Determinations of causality and responsibility are the focus of tort law, in which science may be used to argue that an act of negligence or intent by one party, or a product (e.g., a machine or a chemical) that one party is responsible for, was the cause of loss or harm to another. A demonstration that chemicals introduced into drinking water cause disease is a form of scientific evidence introduced in civil litigation to prove liability (11).

*3) What will happen?*

Proposed actions or products may be seen as legal rights of one party, while another party maintains that those actions/products are a *potential* cause for loss or harm. Here, scientific evidence is introduced to make predictions about the future—to identify causal links and probabilities of things that have not yet happened—and the proposed actions/products enjoined or permitted by a civil court ruling. For example, a home developer that plans to alter the topography of its acreage may be prevented from doing so if the science of hydrology predicts a high probability of water damage to the adjacent property of another party.

*4) Is it new?*

This is the question of patent law. Because patented inventions are often based on scientific principles and technologies, scientific evidence introduced in litigation can be essential for resolving disputes over novelty. This use of science is different from the others in that its purpose is to establish what an invention is, rather than what happened, what was the cause, and who is responsible.

## The Unique Status of Scientific Evidence in the Courtroom

Scientific evidence in the courtroom faces two significant challenges that are less common in other basic research or applied contexts:

*1) The courtroom “user” of scientific evidence lacks understanding or experience*

Unlike physicians and engineers – users of scientific evidence who are educated in scientific methods and principles – those who make decisions in the courtroom based on scientific evidence commonly lack knowledge in the relevant domains. Judges are generalists and juries are routinely composed of laypeople without scientific training. As for other forms of specialized knowledge, scientific experts may be sought to inform the court. There are, however, unique features of science that place unusual demands on an expert.

Science has evolved significantly over the past 200 y, acquiring and refining along the way a treasured set of methods and criteria for controlled investigation of natural phenomena in the search for knowledge. The uniqueness and sophistication of this enterprise, its rigorous requirements for quantification and reproducibility, its intolerance of fraud, and the transparency of the historical record are all characteristics that set scientific research apart from other professions. Scientific evidence is not restricted to the experience of any particular individual; it is drawn from a foundational well of knowledge that is continually deepening and maturing. The scholarly journals of modern science are rich repositories of this knowledge, which has become the basis for decision in many practical ventures, such as medicine and engineering. Because aspects of this science frequently exist outside the realm of experience for courtroom users of the information, the desired role of the expert is to communicate science in plain language to educate the jury. But the scientific expert, unlike the tugboat captain, is not intrinsically the source of that information.

*2) The question demands immediate resolution*

The truth-seeking strategies of scientific investigation and courtroom litigation are both founded on competition and rigorous debate of alternative theories. In practice, however, these adversarial systems operate by fundamentally different rules. Basic science advances through a generative adversarial system, in which disagreement generates testable hypotheses and new discoveries, which can help discriminate between adversarial positions. The result is a progressive accumulation and refinement of knowledge. Law, by contrast, relies upon a terminal adversarial system. Parties in litigation cannot resolve disagreements about cause and effect through further experimentation, as bench scientists might. The battle must be won through existing facts and argument alone.^*^

The problem with the terminal approach is epistemological, at least in part, for it is sometimes difficult to recognize scientific truth. And yet truth – and not just any truth, but *today’s* truth *–* is called for to answer a question of fact.^†^ Karl Popper noted that science risks an “infinite regress” of hypotheses to be falsified, betraying the ephemeral nature of scientific conclusions (12). But Popper also spoke of a “basic statement” that could be made following any empirical test along this path:

“Every test of a theory, whether resulting in its corroboration or falsification, must stop at some basic statement or other which *we decide to accept*… We simply stop when we are satisfied that the piles are firm enough to carry the structure, at least for the time being.”

Scientific evidence presented in litigation may meet Popper’s definition of a basic statement, but the needling question for the gatekeeping judge is always whether the “piles are firm enough to carry the structure.” Ever the pragmatist, Popper concluded that the answer can only be assessed as the “degree of corroboration,” measured not simply by the number of tests but also by “the *severity of tests* to which a theory has been subjected, and the manner in which it has passed these tests, or failed them” (emphasis in original). In practice, corroboration is what yields consensus within the scientific community. The reason that consensus is important can be traced to two fundamental limits of measurement: uncertainty and bias. Leaving aside willful misrepresentation, these are the core reasons why scientific evidence may not be trustworthy. In scientific research, uncertainty and bias are mitigated by empirical validation and reproducibility of results. Validation is the footing for any legitimate application of science because it offers a probabilistic estimate of the accuracy of a measured quantity. To be considered a credible basis for action, however, findings must also be reproducible under different conditions and in the hands of different investigators – yielding a state that Popper termed “intersubjective agreement.” (In simple statistical terms, detection of the same signal by two independent sensors, rather than one, indicates greater likelihood that the signal is real).

In the absence of corroboration, a basic statement presented as scientific evidence simply lacks the empirical support needed to justify a decision. This is a sensible threshold in the practice of scientific investigation, where additional experiments can be performed and alternative hypotheses can be developed and tested, but it hobbles the practice of courtroom litigation where claims can only be vetted by today’s science. Instead, to satisfy fairness constraints, it is often argued that courts should adopt a liberal approach to the admissibility of scientific evidence, allowing jury appraisal of unorthodox views with the conviction that truth will emerge through the adversarial process (13). That conviction may be accurate, but the more difficult situation is when two different scientific interpretations seem plausible and both have significant acolytes, opening the courtroom gate to competing versions of the truth. Generative adversarial science may sort this out eventually, but the terminal approach requires different strategies to limit adverse consequences.

Before considering those strategies, I first summarize current rules for admissibility of scientific evidence to the courtroom, trace how they originated and evolved, and examine how the professions of science and law can collaboratively ensure that courtroom decisions are based on information that can be trusted. Because these topics are deeply embedded in the Law of Evidence, I begin with a brief review of that doctrine.

## The Law of Evidence

The jury system that is a central component of common law was initially based on the belief that members of the community could be self-informed about a local contest and thus serve as both experts and decision-makers (14, 15). All of this changed in the 18th century with the emergence of the Law of Evidence, which understood that complex forms of probative knowledge are often beyond the ken of community jurors. From this understanding emerged two important principles:

1. Jurors should be chosen not for their communal familiarity with contested events but, rather, as blank slates who might be persuaded by credible evidence.
2. Not all evidence is equally credible, requiring that standards be adopted for use.

This transition initiated the instructional approach to jury decisions, in which subject-matter experts are employed to inform juries of the intricacies, context, and meaning of evidence for which they lack the native wisdom and experience to comprehend.

In the early days of this practice, experts were called upon to explain evidence regarding the provenance or authenticity of documents, as, for example, in cases of patrimony or trespass, or to provide medical testimony as to the cause of death (14). Admission of such expert testimony was initially based on informal demonstrations that a) the testimony was relevant and would facilitate jurors’ understanding of complex knowledge (16), “which laymen could not be expected to comprehend and properly estimate” (17), and b) the expert was qualified by education and profession (18). The 19th and 20th centuries saw rapid growth of science and with that came a dramatic increase in the complexity of evidence before the court (19, 20). And so it was that experts from the sciences – physicists, anthropologists, chemists, and biologists – were summoned to opine on probabilities that past events happened as charged or to advise on the likelihood that a faulty machine or toxic substance was the cause of injury (21). Doing so poses a risk, however, that an expert may not have sufficient grasp of the science, may intentionally misrepresent the facts, or that the science itself may not be sufficiently established or relevant. Wary of this threat, the courts began to consider more formal strategies for determining whether scientific testimony is sufficiently trustworthy that it should be presented to the trier of fact.

Because the vehicle for conveying expert legal testimony is the human mind, it seemed natural to look there for the answer. The academic discipline of psychology – the science of mind – sprouted in the mid-19th century from roots in physiology, physics, and philosophy. By the turn of the 20th century, experimental psychology seemed on the cusp of revealing the causes of perception, cognition, and behavior (22). Together with new tools for assessment of nervous system function, psychology promised no less than a full reasoned account of human experience, which would have enormous potential for explaining human decisions in the real world. One of the earliest applications of this new psychology was an assessment of perception and cognition in the context of courtroom decisions, from which a so-called “legal psychology” was born (23). From this auspicious brew emerged the hypothesis that certain measures of brain function might be indicia of deceit, which could be used for assessment of the validity of legal testimony. One of these measures, a precursor to the modern-day polygraph (“lie detector”) test, now lives in legal infamy as the trigger for the first major federal court ruling on admissibility of scientific evidence.^‡^

## The *Frye* Standard for Admission of Scientific Evidence

The first major ruling to effect standards for scientific evidence in the American justice system was handed down one hundred years ago by the US Court of Appeals for the DC Circuit in *Frye v. United States* (24).^§^ James Frye, a twenty-something-year-old resident of the District of Columbia, was questioned regarding the murder of a prominent Washington physician, Robert Brown. Frye confessed to killing Brown accidentally in dispute over payment and was charged with first-degree murder. By the time of his trial in district court, Frye had recanted. His legal defense team had meanwhile recruited the assistance of William Marston, a Harvard-trained psychologist.^¶^ Marston claimed that he could detect deception through changes in systolic blood pressure, elicited by arousal of the autonomic nervous system. Based on laboratory results from this new technique, Marston argued that Frye’s innocence was proved. Skeptical of the scientific foundations and in the absence of “common knowledge” of the method’s validity, the trial court refused to admit testimony based on Marston’s tool (“we do not bring experimental matters into court”) (25). Frye was convicted of the lesser charge of second-degree murder and sentenced to life in prison.

James Frye’s appeal was based on the argument that Marston’s blood pressure machine was indeed a legitimate scientific instrument for assessment of deception. The appeal was heard by the DC Circuit, which sustained the lower court’s ruling that the instrument had not been sufficiently validated:

“We think the systolic blood pressure deception test has not yet gained such standing and scientific recognition among physiological and psychological authorities as would justify the courts in admitting expert testimony deduced from the discovery, development, and experiments thus far made.”

In doing so, the appellate court issued a ruling in 1923 – a ruling nearly as notable for its brevity (669 words) as for its impact (it is among the most heavily cited rulings in American case law) – that ultimately established an admissibility test for scientific evidence. Known today as the “*Frye* standard,” the test was increasingly applied in US Courts for the next seven decades and adopted (and still used) in many state courts as well:

“Just when a scientific principle or discovery crosses the line between the experimental and demonstrable stages is difficult to define. Somewhere in this twilight zone the evidential force of the principle must be recognized, and while courts will go a long way in admitting expert testimony deduced from a well-recognized scientific principle or discovery, *the thing from which the deduction is made must be sufficiently established to have gained general acceptance in the particular field in which it belongs*.” (emphasis added)

Sufficient evidence that the lie detector yielded accurate results simply did not exist, meaning that “the thing from which the deduction [James Frye’s innocence] was made” was neither shown to be valid nor “sufficiently established to have gained general acceptance.” The court thus found that Marston’s instrument remained on the darker side of the twilight zone, where “the evidential force of the principle” had not yet been recognized.

We see in *Frye* the beginnings of a legal formalization of common-sense criteria for the value of scientific evidence, which go well beyond the earlier “helpful” and “qualified” standards. Consider, for example, scientific evidence brought to show that fingerprints found at a crime scene were left there by a suspect in custody. This is evidence of some import. But as a practical matter, how might we know that it is a sound basis for choice? There are several standards common to the sciences for assessing evidence, which are born from laboratory tests conducted under ground-truth conditions:

1. The evidence is obtained using valid methods and is accurate.
2. The evidence is reliable and reproducible by others.
3. There is consensus that the evidence is a sound basis for decisions. *Frye*’s “general acceptance” or consensus standard is based on the premise that a community of people with appropriate expertise could not have come to the same conclusion about the evidence unless the methods were valid and the results accurate, reliable, and reproducible.

## Significant Features of the *Frye* Ruling

There are two significant features of *Frye* that point to the unique status of scientific evidence and the role of the expert:

1. The *Frye* concept of general acceptance is fundamental, as it aligns with Popper’s “degree of corroboration.” Legal theorist Steven Goldberg cited community and communal beliefs as the first of four “Central Dogmas of Law and Science:” “There is in modern America a scientific community capable of forming a consensus on technical matters” (26). Through a distributed process of self-government and policing, this community soundly rejects claims that do not satisfy requirements for validity, accuracy, and reliability. Considered abstractly, if we wish to inform consequential decisions with scientific knowledge, the consensus at any point in time is the most rational ground for doing so. But legal decisions never occur in a social or cultural vacuum; they must be ecumenical and tempered by fairness. All of which encourages flexibility but sets the stage for conflict in the courtroom.
2. The *Frye* ruling refers only to the character of the “scientific principle” proffered as evidence. *Frye* makes no mention of subject-matter expertise (27), except in the sense implied by the “general acceptance” clause, which refers to the *consensual expertise* held by the scientific community. This concept of consensual expertise as the basis for establishing trustworthiness became eroded through establishment of the Federal Rules of Evidence, which turned the focus toward opinions of individual experts.

## Rule 702: Testimony by Experts

By the 1970s, a sense had emerged that the inflexible *Frye* requirement for general acceptance was difficult to establish and perhaps insufficient, in that it was mainly relevant to criminal cases in which an invented instrument was proposed to establish fact.^#^ Partly in response to this concern, standards for admissibility of scientific evidence began to change. They did so initially, at least in a formal sense, following recommendations of a federal advisory committee of the United States Judicial Conference, which was established for the broader purpose of normalizing and codifying rules for the use of evidence in US Courts. The Federal Rules of Evidence became law in 1975 by act of Congress. The particular rule that bears on admissibility of expert testimony is known as Rule 702 (28).

While *Frye* selectively targets the use of scientific evidence, Rule 702 applies more generally to expert testimony on “scientific, technical, or other specialized knowledge,” meaning that the same standards apply to evidence drawn from the well of scientific knowledge and to subject-matter experts in nonscience knowledge domains, such as tugboat captaining. In its original form, Rule 702–1975 merely formalized and made into law standards for “helpfulness” and “expert” qualifications, both of which had been less formally applied since the 19th century:

“If scientific, technical, or other specialized knowledge will assist the trier of fact to understand the evidence or to determine a fact in issue, a witness qualified as an expert by knowledge, skill, experience, training, or education, may testify thereto in the form of an opinion or otherwise.”

As a notably limp standard for judging the evidentiary nuances of modern science, Rule 702-1975 was subsequently interpreted and clarified by the Supreme Court’s transformative 1993 ruling on scientific evidence in *Daubert v. Merrell Dow Pharmaceuticals, Inc.* (8).

## The *Daubert* Standard

Unlike the forensic instrument that motivated the *Frye* standard, in which the question before the court concerned the scientific validity of measured quantities, the *Daubert* ruling emerged from a toxic tort case, in which scientific evidence attempted to establish cause and effect. Daubert’s civil action was filed against the drug company Merrell Dow Pharmaceuticals in 1984, on behalf of two children born with serious birth defects. The mothers had taken the drug Bendectin [doxylamine succinate and pyridoxine hydrochloride (vitamin B_6_)], which was manufactured by Merrell Dow and widely used for decades to quell nausea and vomiting during first-trimester pregnancy. The plaintiff alleged that Bendectin had caused deformities during gestation. Merrell Dow maintained that there was no scientific evidence of a link between their drug and birth defects, but Daubert recruited an expert in the form of obstetrician William McBride, who was prepared to testify on the teratogenic effects of Bendectin. Noting that McBride’s assertions failed to meet the *Frye* standard of general acceptance by the scientific community, the District Court for the Southern District of California issued summary judgement in favor of Merrell Dow (29). Daubert appealed to the Ninth Circuit, which upheld the lower court’s ruling (30).

In response, Daubert went on to argue before the Supreme Court that the common law *Frye* standard for admissibility of scientific evidence was inapplicable in their case, because it had been replaced in 1975 by the legislatively enacted Rule 702. The Court agreed and upheld Rule 702–1975 as the modern legal standard for admissibility in federal court, superseding the *Frye* standard.^‖^ In its ruling (8), the Court provided an interpretation of Rule 702–1975, which is known today as the *Daubert* standard. This standard consists of a set of clear and useful criteria for assessing the trustworthiness of scientific evidence:

• whether the theory or technique in question can be (and has been) tested,
• whether it has been subjected to peer review and publication,
• its known or potential error rate, and
• the existence and maintenance of standards controlling its operation, and
• whether it has attracted widespread acceptance within a relevant scientific community
Unlike the uncompromising *Frye* standard, these criteria are intended to be flexibly applied at the discretion of the trial judge.

With these brief considerations, *Daubert* strengthened the application of evidence law in several ways that conform to the nature of scientific investigation. Perhaps most importantly, *Daubert* returned the focus to the body of scientific knowledge (27), highlighting the importance of empirically demonstrating [“can be (and has been) tested”] that a scientific instrument or principle is a valid predictor of the probability that a courtroom hypothesis is correct (“known or potential error rate”). To that end, the focus on widespread or general acceptance of scientific evidence – consistent with *Frye* but absent from Rule 702 – is notable here, as the scientific consensus at any moment is the rational basis for decision under the unyielding demands of courtroom litigation. Also consistent with *Frye* and contrary to the letter of Rule 702, *Daubert* emphasizes the need for evidence to reflect the consensus of the “relevant scientific community.” As highlighted below, the definition of relevance has become a battleground in efforts to reform the use of forensic evidence.

## Rule 702 Evolves

Rule 702 was substantially amended in 2000 to conform with *Daubert* and to promote a “more rigorous and structured approach” (31), in which the gatekeeping role was formally handed to judges. The Rule’s emphasis on the expert remained, but three “reliability” requirements were included in Rule 702–2000 (provisions b-d), which place constraints on the data, methods, principles, and their application by the expert:

A witness who is qualified as an expert by knowledge, skill, experience, training, or education may testify in the form of an opinion or otherwise if:

(a) the expert’s scientific, technical, or other specialized knowledge will help the trier of fact to understand the evidence or to determine a fact in issue;
(b) the testimony is based on sufficient facts or data;
(c) the testimony is the product of reliable principles and methods; and
(d) the expert has reliably applied the principles and methods to the facts of the case.

Rule 702 was revised again in 2022 (to take effect December 2023) through amendments proposed by the Advisory Committee on Evidence Rules and subsequently approved by the US Judicial Conference and the Supreme Court (32). For these efforts, Rule 702–2022 differs from the previous by two small text additions. One defines a preponderance of evidence (“more likely than not”) standard for demonstrating that the four provisions [702(a-d)] have been satisfied, which offers the gatekeeping judge a quantitative criterion for decisions about admissibility. The other clarifies that it is not the expert’s reliable application that matters, but rather that “the expert’s opinion reflects a reliable application,” which grants the trial judge the ability to bar opinions that exceed what can be reasonably concluded from the methods and principles applied.

In the following paragraphs, I discuss some of the strengths and weaknesses of the Rule, post *Daubert*, as seen in the light of evidence standards routinely employed by the scientific community.

• Although Rule 702 promotes a healthy focus on the validity of scientific evidence, it turns the emphasis away from the *Frye* “general acceptance” standard and toward attributes of the expert. This partly reflects the fact that it was designed as a general-purpose rule for experts with specialized knowledge, not simply scientific experts. In doing so, however, the Rule fails to highlight scientific consensus, which is a primary determinant of trustworthiness. While consensus is appropriately recognized by *Daubert* via the “widespread acceptance” criterion, it should not be discretionary; consensus based on corroborative evidence is how we know things are true.
• The introduction sets a loosely defined bar for qualifications of the expert. Lost from *Frye*, and failing to heed *Daubert*, is the requirement that the expertise arise from the relevant scientific community (“the particular field in which it belongs”). Although the relevant community is sometimes difficult to resolve from a viewpoint outside of the scientific enterprise, it would help to formally distinguish experts who opine on what a scientific instrument (or method, or test) does vs how well it works.^**^
• 702a is the helpfulness criterion. Neither the introduction nor 702a speak to qualifications for the communication/education role of the expert, which is a prerequisite for helpfulness when conveying complex evidence. The focus should be placed on the expert’s ability to clearly communicate the relevant science to a lay audience. While parties may apply their own criteria for communication skills, the effect is sometimes obfuscation (33), which highlights the importance of courtroom standards.
• 702b and 702c define attributes of the evidence itself that qualify it for admission. The emphasis on “sufficient facts or data” to support all facets of the expert’s testimony (702b) is critical to any rational decision based on scientific knowledge. Marston’s lie detector was excluded simply because there were very few facts or data to support claims of validity. The requirement that evidence be a “product of reliable principles and methods” (702c) is also critical. Evidence presented in support of a hypothesis should be empirically validated to reveal the probability that the hypothesis is true. Reproducibility—demonstration that evidence holds up across studies—is also important to overcome errors and biases that may be unique to a given study, and to gain consensus (12).
• Finally, 702d is a requirement that expert opinions not exceed what is possible to conclude given the knowledge base and methods employed – the premise being that, once admitted, a lay jury may not be able to identify false or exaggerated conclusions drawn from complex evidence. Among the important targets of this provision is a tendency to generalize, without evidence, from scientific conclusions about group effects to conclusions about individual cases (10).

The positive impact of *Daubert* on the structure of Rule 702 cannot be overstated. It also continues to complement and clarify application of the Rule. Many courts today adopt a hybrid approach, in which the sensible standards of *Daubert* are used to bridge gaps in Rule 702. The problematic status of the “expert” nonetheless remains, with far-reaching implications for justice.

## Are Experts Important?

I have argued that empirical demonstration of validity and reproducibility, and development of consensus within the relevant community, should be the primary criteria for admissibility of scientific evidence. Rule 702 nonetheless specifies criteria based on an expert’s scientific bona fides and ability to assist the trier of fact. This distinction raises an interesting empirical question: What is more persuasive to the trier of fact, the quality of the science or the attributes of the expert? Recent evidence suggests the latter (34). One might write a timely thesis on the cultural prominence of person over fact, but this is the common law system we live with today. Here, I discuss other attributes of experts and their potential impact on the justice system in which they serve.

## Who Gets to be an Expert?

The “general acceptance” clause in *Frye* maintains that conclusions about consensus on a specific principle or discovery must be drawn from “the particular field in which it belongs.” The “widespread acceptance” clause in *Daubert* similarly states that acceptance must exist “within a relevant scientific community.” While it would be hard to argue against these directives, a persistent sticky point has been: What is the relevant scientific community? (35).

Distinctions between scientific disciplines are not in question here. The opinions of a herpetologist are scarcely relevant to fingerprint analysis. But there is a different way of parsing expertise that comes up frequently in applied sciences: the distinction between a *researcher* and a *practitioner* of scientific principles and techniques. The researcher is, by definition, the person involved in scientific experimentation and discovery, who has acquired an understanding of scientific principles of relevance to a real-world problem. The practitioner is, by contrast, the person with knowledge of the prevalence and characteristics of a problem in the real world who employs science-based inventions to mitigate the problem. In medicine, for example, the researcher is a basic scientist who has discovered the ameliorative effects of a chemical compound, which is made into a drug. The practitioner is the physician who prescribes the drug to a clinical population.

Both practitioners and researchers have domain-specific knowledge that may inform the court in different ways. Consider, for example, the respective contributions of the researcher and practitioner to questions of forensic firearms identification. If the court wishes to know something about the physical process by which firearms leave marks on shell casings, or the method that is used by a forensic lab to locate and characterize such marks, then the forensic practitioner is the best person to consult. If, on the other hand, the court wishes to know *how well* a forensic process is performed, the best person to consult is a basic scientist with expertise in human factors that affect the validity of human sensory decisions (36). Justice may be better served by clarifying this distinction when considering admissibility of scientific evidence.

## Terminal Adversaries, Partisan Experts, and the Fracturing of Scientific Knowledge

*“Our courts are confronted with a pervasive phenomenon that often approaches and sometimes crosses the line into the realm of scandal.”* (37)

In consideration of due process, the procedures for courtroom litigation in American common law permit each party to recruit their own experts to convey scientific evidence in support of their position. In some ideal world, perhaps, these experts would present complementary details around the scientific consensus of the day, which may be advisory of one path or another, much like getting a second opinion on a root canal, or how to best repair the truck. This system fails in the legal context, however, because the parties are in conflict over the outcome, which means that the scientific experts they hire bear commitments that may place them in conflict with the truth. The result is a proliferation of testimony tagged disparagingly as “junk science,” sometimes proffered with the intent to introduce uncertainty or obfuscate (33).

This is hardly a new observation (27, 38). The case of *Keegan v. Minneapolis & St. Louis Railroad*, argued before the Minnesota Supreme Court over 120 y ago, followed from a successful civil action brought by the family of a traveling salesman who died of endocarditis following a fall from a transport carriage. That fall was precipitated by a road that was poorly maintained by the defendant. In trial court, the plaintiff and defendant presented dueling experts opining on the causal relationship between the injury and the terminal disease, which the jury was forced to reckon with for judgment. The Minnesota Court upheld the lower court’s ruling, and in its wisdom broadly condemned the pathology of our judicial system that fosters competing versions of scientific truth:

“Experts are nowadays often the mere paid advocates or partisans of those who employ and pay them, as much so as the attorneys who conduct the suit. There is hardly anything, not palpably absurd on its face, that cannot now be proved by some so-called ‘experts (39).’”

This pathology certainly caught the attention of jurist Learned Hand, who famously wrote:

“Enough has been said elsewhere as to the natural bias of one called in such matters to represent a single side and liberally paid to defend it. … The result is that the ordinary means successful to aid the jury in getting at the facts, aid, instead of that, in confusing them… How can the jury judge between two statements each founded upon an experience confessedly foreign in kind to their own?” (14).

Indeed, how can they? In her brilliant analysis of the role of science in legal matters, Sheila Jasanoff cut straight to the underlying problem of choice in this context:

“the formal and adversarial style of regulatory decisionmaking highlights uncertainty, polarizes scientific opinion, and prevents efficient resolution of disputes about risk. Far from promoting consensus, knowledge fed into such a process risks being fractured along existing lines of discord” (40).

Substitute “courtroom litigation” for “regulatory decision-making” and “disputes about cause” for “disputes about risk,” and the statement precisely captures the dysfunction with scientific testimony in American courts. This problem is well recognized in many scholarly texts on legal policy, and often expressed with sarcasm: Trials are “increasingly similar to barking seal contests in which rival trainers compete to induce their teams of trained experts to make the most winsome noises” (37). The advisory committee for the Federal Rules of Evidence considers “the practice of shopping for experts” a “matter of deep concern” (41). More recently, this phenomenal splitting of expert testimony along party lines has shown up in experimental results as not simply an overt effort to support a client, but as an unconscious yet quantifiable shift in an expert’s opinions and interpretation of facts – “working for one side in an adversarial case causes some experts’ opinions to drift toward the party retaining their services” – a cognitive process known as “adversarial allegiance” (42).

This “matter of deep concern” is where the difference between generative and terminal adversarial systems hits home. Decision-making – in scientific research and in the courts – depends on the ability to evaluate the relative probabilities that different hypotheses are true, given the evidence at hand. In science, when evidence is equivocal we generate another empirical test with the belief that the new evidence gained will tip the scale.

In courtroom litigation, by contrast, we paint lipstick on the pig, because she’s all we’ll ever have. And in the process, we fall in love with her. This is not the rational approach to fateful decisions that any scientist would advocate. If the evidence appears equivocal and the process is ineludibly terminal, the smart thing is to take a much closer look at the evidence, not dress it up with partisan “experts.” Doing so necessarily requires a broader engaged collaboration between the courts and the scientific community. To that end, there are several strategies worth considering.

### Duty to the Court?

One approach to this vexing problem would be to transfer duty of the expert from the parties to the court and shore up the process by which the court can determine that the expert is correctly channeling the scientific consensus. Following the recommendations of a UK commission empaneled in the 1990s by Lord Harry Woolf (later Chief Justice of England and Wales), which noted the obvious problem – “experts had become partisan advocates rather than neutral givers of opinion” (43) – the courts in England and Wales revised procedural rules. “The basic premise of these [new] rules is that the expert’s function is to help the court, not to advance the case of the side by whom he or she is paid” (43). By this astute argument, scientific experts are appointed either through mutual agreement of the parties or at the discretion of the court. Their duty is “owed to the court” and “overrides any obligation to the person from whom experts have received instructions or by whom they are paid” (44).

On the surface, this neutral advisory function is antithetical to the adversarial system of law practiced in the United States. But it does seem to work in other common law countries, and the pitfalls of adversarial allegiance undermine the credibility of our existing judicial system. The Federal Rules of Evidence do include a provision for court-appointed experts (Rule 706) (45), which has had some success in educating courts on scientific matters (46). The mechanism is rarely used, however, in part because it stands in awkward conflict with the adversarial process (47). That awkwardness stems from a courtroom culture that empowers a competitive marketing approach to decision-making – indeed, litigators express “a marked preference for retained experts” (48) – while neglecting the view that truth and fairness in the courtroom, and increased accuracy of decisions, can only come from deeper understanding of the evidence.

### Recognizing Scientific Consensus.

Deeper understanding of evidence in this context requires impartial review and scrutiny to assess the probability that one or another hypothesis is true. In scientific investigation, this is the function of peer review. Many established journals with blind peer review have extremely high standards for publication and scientific work therein is commonly read and cited with high frequency. While this offers an approximation of scientific consensus, unexpected findings and minority views are sometimes shunned by this elite process and forced to publish in fringe journals, causing critics to argue that the system mainly supports the status quo (49). In *Dauber*t, the Supreme Court noted that petitioners and their *amici* expressed precisely this concern: “exclusion of ‘invalid’ evidence will sanction a stifling and repressive scientific orthodoxy and will be inimical to the search for truth.” *Daubert* maintains, furthermore, that “Publication…is not a sine qua non of admissibility; it does not necessarily correlate with reliability.” The scientific community, by contrast, adopts a probabilistic view, arguing that publication through peer review correlates with validity but does not *guarantee* it (50). Nonetheless, if we accept that science published in high-quality journals is not necessarily valid and, conversely, valid science does not always receive the publication status it is due, then how can our courts possibly identify science that is both valid and generally accepted? One solution is to adopt the strategy that the scientific community generally employs for decisions about funding, promotions, and research priorities, which is based on a broader deliberative and open review of relevant science.

### Science Courts and Scientific Peer Review Panels?

To this end, a controversial and long-neglected (51) proposal is to form a “Science Court” in which scientific experts for the parties “direct their best arguments at each other and at a panel of sophisticated scientific judges” (52). The concept is that scientific judges are prepared, unlike a traditional judge, to vet complex scientific evidence, such that they can supply the trial court with “statements founded on that knowledge, which will provide defensible, credible, technical bases for urgent policy decisions.” In effect, the science court would serve as a gatekeeper for scientific expert testimony and would package the product of its review as the consensus of the time for use by the trial court. The concept is similar to use of standing scientific committees that inform decision-making by regulatory agencies. The science court idea does have its detractors, however, who note a) evaluation of science removed from the larger context of a legal case may fail to consider contraindications or practical limitations from the real world, and b) a standing science court places too much power in the hands of too few.

An attractive alternative exists in the form of ad hoc peer review panels, or Consensus Panels (53), of the sort commonly convened by funding agencies to evaluate specific areas of proposed scientific research. Rotating panels would be embedded within the evidentiary process and aim to identify the scientific consensus regarding expert testimonies from the parties and advise the trial judge, all within the same pre-trial hearing.

To ensure fairness, a science court or a science advisory panel must not preclude input from traditional partisan experts (54). These scientific review strategies are nonetheless likely to be beneficial because debate is overseen by scientific experts – not judges – who a) have no investment in the outcome, b) are qualified to zoom-in closely on the evidence, and (c) can call-out exaggeration of the relevant science, or generalization beyond the bounds of the underlying principles and methods. Practicing scientists will recognize this as the same peer review process that keeps scientific research in check by adjudicating claims in a search for knowledge and certainty. A related approach, known as concurrent expert testimony, or “hot tubbing,” encourages experts for the parties to work together and identify areas of agreement and dispute. However sensible this latter approach may seem, there is little evidence that it works to reduce adversarial allegiance, possibly because there is no oversight and moderation by an independent body of scientists (55, 56).

### Severing Partisan Ties Without Sinking the Ship.

Real adversarial allegiance in expert testimony, or suspicion of it on the part of the trier of fact, is heavily influenced by the act of retention (57). The alternatives considered above – duty to the court or the use of impartial scientific review panels – are promising precisely because they sever those partisan ties. But they are not free, either with respect to time or value of specialized knowledge. The expense must come from a source that has no investment in the outcome. In their current incarnations, our courts cannot afford this. But as a free society, we live by the principle of unrestricted access to unbiased information. Our modern movement toward “open science” – public access to the corpus of publicly funded science, uncontrolled by source and unvarnished by partisan interests – is a start. In the same spirit, scientific societies could adopt professional rules of conduct that promote awareness of ways in which science is used by the courts, discourage adversarial allegiance by their members, and encourage legal testimony that hews closely to the scientific consensus. Some professional societies, such as the American Psychological Association and the American Academy of Psychiatry and the Law, do have professional guidelines that apply to legal testimony. While these guidelines stress impartiality, their primary focus is on privacy.

## A Path Forward: Cross-Pollinating Science and Law

The phenomenal growth of modern science and the demands placed on scientific evidence in courtroom litigation raise important questions about the definition of scientific truth and how to recognize it in “terminal” applications. These questions have been partially addressed by legislative and judicial actions, but there are still holes to be filled. Adversarial allegiance and polarization of scientific opinion on questions of fact are predictable and highly dysfunctional consequences of a free market approach to information in an end-of-days courtroom economy. Such crises can be avoided by greater consilience between the disciplines of science and law, and the establishment of close working relationships that are independent of the interests of parties seeking specific legal actions. To this end, it is useful to view law as an applied science, as it pertains to questions of fact. To illustrate the benefits of this perspective, consider medicine, which is one of the most successful applied sciences of our time. In the United States today, there is a highly regulated process for assessing the validity of drugs through clinical trials (58). This process ensures that fateful decisions about drug use are based on high standards of evidence, and it is successful at doing so because of the close intellectual partnership between scientists and physicians. Bad science in the courtroom could be mitigated by similar efforts to cultivate close working relationships between scientists and legal professionals.

Such efforts are not without precedent, as seen through the work of both governmental and independent commissions and task forces (59, 60, 61). Many academic institutions also now house thriving centers operating at the interface of science, legal policy, and practice. The Committee on Science, Technology, and Law (CSTL), of the National Academy of Sciences (NAS), is among the most successful advisory panels convened for this purpose. CSTL was conceived to address “the evergrowing need for the legal and scientific communities to work with each other on issues of importance to the nation. The need for a prominent forum for representatives of these communities to get to know each other, understand their cultures, and exchange ideas” (62). CSTL has overseen production of consensus reports on a range of topics at the intersection of science and law, including forensic science (60), eyewitness identification (63), and the highly valued *Reference Manual on Scientific Evidence* (64). CSTL’s products are a respected source of guidance and its process is a grass roots model for the larger science-law culture that we envision.

## “The Law Desires Truth, but Realistically Settles for Justice and Fairness” (65)

Despite our best efforts, there will always be tension between a) the scientists’ ideal that momentous decisions be based upon the highest standards of evidence, b) the practical reality that those standards are sometimes difficult to meet, or that relevant science does not exist, and c) our unassailable right to fairness. This tension is workable, in principle, through compromise, flexibility, and well-reasoned understanding of criteria for scientific truth, but it forms a leaky structure that is easily exploitable, particularly in our age of misinformation. The solution is to promote awareness and vigilance by all parties and to foster collaboration between gatekeeping judges and scientists. “As the knowledge of science and the procedures of law evolve, the need for this ‘cross-pollination’ becomes ever more necessary” (62).

## Data Availability

There are no data underlying this work.

## Footnotes

^*^The exception is “litigation-generated science” in which a party commissions a scientific study to answer a question of fact raised in ongoing litigation. There are no coherent statistics on the frequency of this practice, but it is believed to be rare because of time and expense, and discouraged because outcomes are susceptible to bias.

^†^Many argue that the concept of a singular scientific truth is naïve. This existential argument is irrelevant to the court, where the question of truth is more mundane and probabilistic: What current information has the highest likelihood of yielding a correct judicial outcome?

^‡^The fascinating backstory to this ruling, complete with subterfuge, intrigue and academic scandal, is well told by historian Jill Lepore (25).

^§^In 1911, *People v. Jennings* (252 Ill. 534, 549, 96 N.E. 1077) allowed fingerprint evidence on grounds of general acceptance and qualified expertise. While this state court ruling ushered in the use of fingerprints in criminal trials, it did not have *Frye*’s broader impact.

^¶^The same William Marston who created Wonder Woman.

^#^Prior to the 1970s, which saw an increase in toxic tort claims, *Frye* had no appreciable role in civil litigation.

^‖^The *Frye* standard remains in effect today in several state courts.

^**^A pervasive problem with some forms of forensic evidence, as discussed below.
